# SARS-CoV-2 Molecular Network Structure

**DOI:** 10.3389/fphys.2020.00870

**Published:** 2020-07-10

**Authors:** José Díaz

**Affiliations:** Laboratorio de Dinámica de Redes Genéticas, Centro de Investigación en Dinámica Celular, Universidad Autónoma del Estado de Morelos, Cuernavaca, Mexico

**Keywords:** SARS-CoV-2, virus interactome, network topology, scale-free network, therapeutic targets

## Abstract

Knowledge about the molecular basis of SARS-CoV-2 infection is incipient. However, recent experimental results about the virus interactome have shown that this single-positive stranded RNA virus produces a set of about 28 specific proteins grouped into 16 non-structural proteins (Nsp1 to Nsp16), four structural proteins (E, M, N, and S), and eight accessory proteins (orf3a, orf6, orf7a, orf7b, orf8, orf9b, orf9c, and orf10). In this brief communication, the network model of the interactome of these viral proteins with the host proteins is analyzed. The statistical analysis of this network shows that it has a modular scale-free topology in which the virus proteins orf8, M, and Nsp7 are the three nodes with the most connections (links). This result suggests the possibility that a simultaneous pharmacological attack on these hubs could assure the destruction of the network and the elimination of the virus.

## Introduction

SARS-CoV-2 has infected over 11,000,000 people since the end of 2019 and killed about 500,000 people worldwide. The SARS-CoV-2 virion is formed by four proteins: the envelope protein E, the membrane protein M, the nucleocapsid protein N, and the spike protein S. A detailed review of the structure of these proteins can be found in the work of McBride and Fielding (2012). Infection begins when the protein S of the virion binds with high affinity to the cell-surface receptor ACE2 (Angiotensin-Converting Enzyme 2), which is highly abundant in lung alveolar type II cells (Hamming et al., 2004). The formation of the complex S-ACE2 initiates the process of fusion between the virion envelope and the cell membrane, leading to the liberation of the nucleocapsid with the viral genome into the cytoplasm (Letko et al., 2020; Walls et al., 2020).

**Figure 1:**
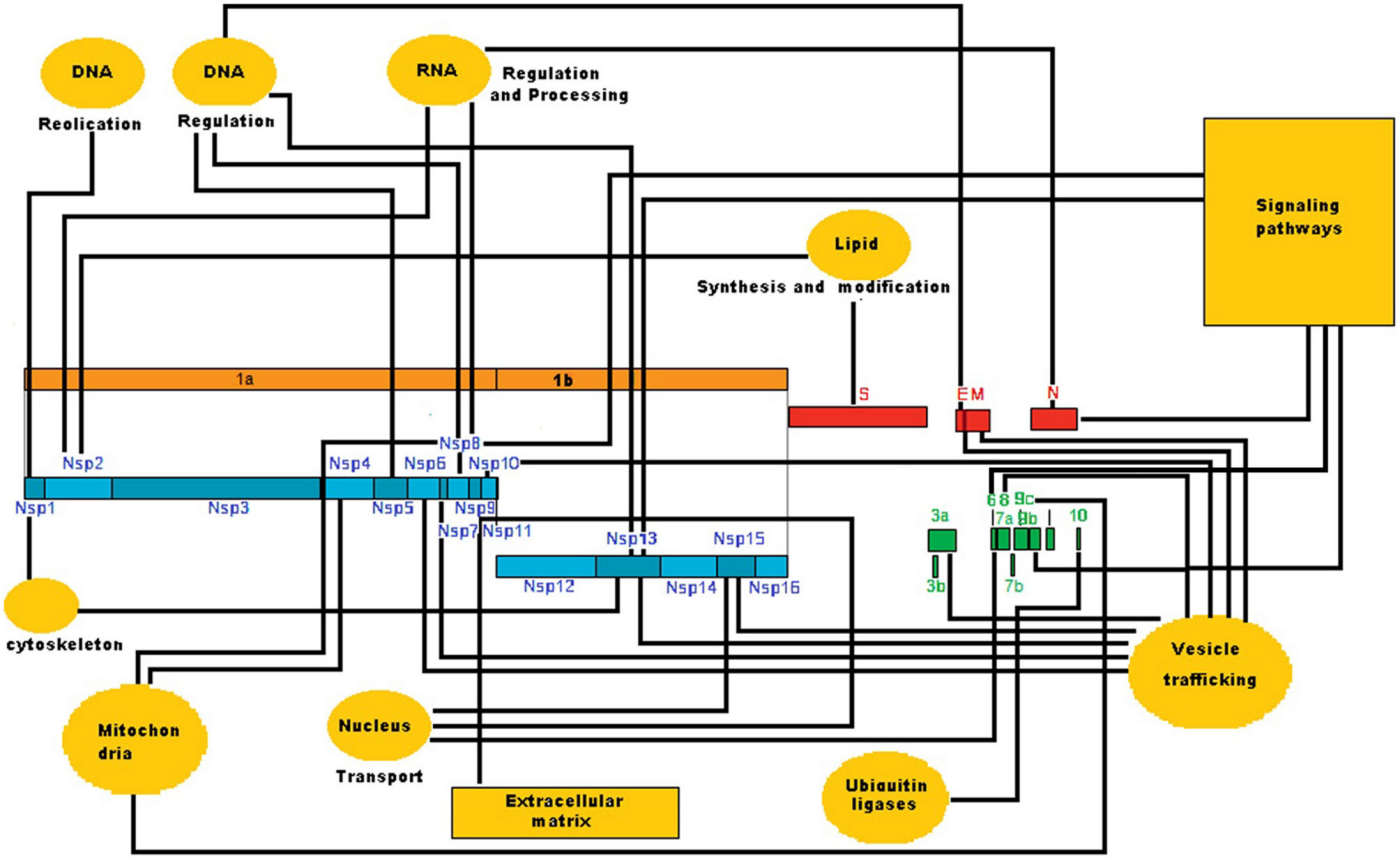
Interaction of viral proteins with host processes in SARS-CoV-2 infection. This is a circuit representation of the production of the viral proteins and the host processes targeted by them. The circuit shows that viral proteins mainly alter the host vesicular trafficking process. Viral proteins also modify other host processes such as signaling pathways and RNA processing, among others. The circuit was drawn from the experimental data of Gordon et al. (2020).

The SARS-CoV-2 genome consists of a positive-sense non-segmented single-stranded mRNA [(+)ssRNA] of ~ 30 kb. The open reading frames 1a (orf1a) and 1b (orf1b) are located near the 5′UTR of the (+)ssRNA, and they code for the polyproteins pp1a and pp1ab. Maturation of these polyproteins results in 11 non-structural proteins (Nsp) from the orf1a segment (Nsp1 to Nsp11) and five non-structural proteins from the orf1b segment (Nsp12 to Nsp16). Nsp proteins form the replication-transcription complex (RTC) in a double-membrane vesicle where a set of nested subgenomic minus-strands of RNA [(–)sgRNA] are synthesized in a process of discontinuous transcription. These (-) sgRNAs serve as the templates for the production of subgenomic mRNAs from which the structural proteins E, M, N, and S, together with the accessory proteins orf3a, orf6, orf7a, orf7b, orf8, orf9b, orf9c, and orf10 are synthesized (Sevajol et al., 2014). The information for the production of these proteins is coded in the one-third of the viral genome near the 3′-UTR. A more detailed description of the function of each viral protein is presented in the work of McBride and Fielding (2012). Viral proteins interact with the molecular machinery of the host cells, take control of it, and redirect the host activity toward the production of more virus particles (Masters, 2006). Figure 1 shows the host functions that are altered by the presence of this set of proteins (Masters, 2006; Gordon et al., 2020; Wu et al., 2020).

Network theory is a powerful tool for understanding the organization and dynamics of complex systems (Breitling, 2010). A theoretical approach to biological network structure and function allows the integration of dispersed experimental data into a coherent model of the spatiotemporal dynamics of interconnected cellular processes. In virology, a model of the dynamics of the gene regulatory network (GRN) of HIV-1 has recently been published (Bensussen et al., 2018). The model is used to analyze the form in which HIV-1 utilizes latency to evade the cellular mechanisms that can destroy it. Results obtained from this analysis were used to propose some therapeutic agents with probable clinical application against HIV-1.

In this sense, the application of network theory to the analysis of SARS-CoV-2 infection could be a guide for the design and use of antiviral drugs against specific viral proteins. However, due to the novelty of this virus species, there is a lack of the quantitative information necessary to propose an ODE-based continuous model for the analysis of the spatiotemporal dynamics of the infection. However, Gordon and collaborators (Gordon et al., 2020) cloned, tagged, and expressed 26 viral proteins in human cells using affinity-purification mass spectrometry to identify the human proteins physically associated with each each viral protein. They found ~332 SARS-CoV-2-human protein–protein interactions that form the virus interactome. This result allows the construction of a viral network representation of the interactome and its statistical analysis.

Thus, the objective of this brief communication is to publish the results obtained from statistical analysis of the SARS-CoV-2 network. These results suggest the possibility that the network has a hierarchical scale-free modular structure with three highly connected viral proteins or hubs (orf8, M, and Nsp7) that control most of the cellular processes (Figure 1). As a consequence, the present work proposes the hypothesis that, to defeat the infection, the combination of at least three different medicaments is necessary for a successful pharmacological attack on these hubs.

## Methods

The SARS-CoV-2 undirected network was built by using the qualitative experimental data obtained by Gordon et al. (2020) and additional data reported in the literature (Masters, 2006; Wu et al., 2020). Gephi 0.9.2 was used to do most of the statistical analysis of the network and to draw its graphical representation with the Fruchterman-Reingold algorithm (Fruchterman and Reingold, 1991). Supplementary statistical analysis was performed with Biostat ver. 5.8.4.3 (2010) and NCSS Data Analysis Software (2020).

The basic statistical analysis of the network includes the determination, for each node, of the value of the following parameters: degree, clustering coefficient, closeness centrality, betweenness centrality, and modularity class. The average clustering coefficient and the degree distribution of the complete network were also calculated. Table 1 summarizes the values of the degree, clustering coefficient, and modularity class for the top 25 most connected nodes. Table S1 shows the values of all of these parameters for each node of the complete network.

**Table 1:** Basic statistical analysis of the SARS-CoV-2 network and FDA-approved drugs.

**Protein**	**Degree**	**Clustering coefficient**	**Modularity class**	**FDA-approved drugs**
orf8	47	0.00185	13	Rapamycin, FK-506
M	29	0.002463	19	Bafilomycin A1?
Nsp7	26	0.003077	3	Entacapone, Indomethacin, Metformin
orf9c	23	0.003953	15	Haloperidol, Metformin, Daunorubicin, S-verapamil
Nsp12	21	0.004762	6	Ponatinib
Nsp13	20	0.005263	8	
Nsp8	19	0.040936	4	
Nsp9	11	0	0	Dabrafenib
orf1a	11	0	2	
orf9b	11	0	14	Midostaurin, Ruxolitinib
Nsp2	8	0	2	Rapamycin
orf3a	8	0.035714	9	
N	7	0	18	Rapamycin, Silmitasertib
Nsp4	6	0	2	
orf10	6	0	16	
E	6	0	17	JQ1
Nsp10	5	0	5	
Nsp1	4	0	2	
Nsp5	4	0	2	Valproic Acid
Nsp14	4	0	1	Migalastat, Mycophenolic acid, Ribavirin
Nsp15	4	0	1	
orf1b	4	0	1	Remdesivir?
Nsp6	3	0	2	Haloperidol, Chloroquine
orf6	3	0	11	
NGDN	3	1	4	

The number of links of a node *N*_*i*_ to its neighbor nodes defines its degree *k*_*i*_. The degree distribution of an undirected network is defined as the number of nodes with degree *k* (*m*_*k*_) divided by the total number of nodes *m*:

(1) $$\begin{array}{r} P\left(k\right)=\frac{m_{k}}{m} \end{array}$$

*P*(*k*) is a probability distribution where *k* = 0, 1, 2,… and $\underset{k}{\sum }P\left(k\right)=1$. In random networks, *P*(*k*) is a binomial distribution, while in scale-free networks, *P*(*k*) is a decaying exponential. In the last case, *P*(*k*) obeys a power-law distribution:

(2) $$\begin{array}{r} P\left(k\right)\sim k^{-\gamma } \end{array}$$

where the exponent γ has a value between 2 and 3. This power-law property is independent of the size (scale) of the network and indicates that a few nodes or hubs determine the connectivity of the network, establishing a hierarchical form of organization.

For a node *N*_*i*_ with *l* links with its neighbors in an undirected graph, the clustering coefficient is defined as:

(3) $$\begin{array}{r} C_{i}=\frac{2l}{k_{i}\left(k_{i}-1\right)} \end{array}$$

where *C*_*i*_ represents the density of links associated with node *N*_*i*_, i.e., the proportion of links between node *N*_*i*_ and its neighbors divided by the number of links that could possibly exist between the neighbors.

The average clustering coefficient of the network with *m* modes is defined as:

(4) $$\begin{array}{r} C_{N}=\frac{1}{m}\sum_{i=1}^{m}C_{i} \end{array}$$

which is simply the average of the clustering coefficient of each node *N*_*i*_.

Modularity is the fraction of links that fall within a cluster minus the expected fraction if links were distributed at random. Modularity indicates the nodes that are more densely connected with each other than with the rest and reveals clues about the structure and the vulnerable spots of a network. The modularity of a non-random network has a value between 0 and 1. The software Gephi uses the Louvain method for community detection:

(5) $$\begin{array}{r} Q=\frac{1}{2m}\underset{i,j}{\sum }\left[A_{i,j}-\frac{k_{i}k_{j}}{2m}\right]\delta \left(c_{i},c_{j}\right) \end{array}$$

where *Q* is the modularity of the network, *A*_*ij*_ is the weight of the link between node *i* and node *j*, $k_{i}=\underset{j}{\sum }A_{ij}$ are the weights of the links attached to node *i, c*_*i*_ is the cluster to which node *i* is assigned, and $m=\frac{1}{2}\underset{i,j}{\sum }Aij$. In Equation (5), δ(*c*_*i*_, *c*_2_) = 1 if *c*_1_ = *c*_2_, and 0 otherwise (Blondel et al., 2008). Gephi finds the modularity classes of a network with an algorithm that compares the number of links *among clusters* to the number of links expected in a random network.

The closeness centrality of a node (σ_*i*_) is a measure of centrality in a network, calculated as the reciprocal of the sum of the length of the shortest paths (geodesic paths) between node *i* and the rest of the nodes in the graph (Newman, 2010). Thus, the more central a node is, the *closer* it is to all other nodes.

Denoting by *d*_*ij*_ the length of the geodesic path between node *i* and node *j*, the mean shortest distance from *i* to *j*, averaged over all nodes *j* of the network (*n*) except *i* itself, is:

(6) $$\begin{array}{r} L_{i}=\frac{1}{n-1}\underset{j}{\sum }d_{ij} \end{array}$$

Thus, the closeness centrality of node *i* is:

(7) $$\begin{array}{r} \sigma_{i}=\frac{1}{L_{i}}=\frac{\left(n-1\right)}{\underset{j}{\sum }d_{ij}} \end{array}$$

Betweenness centrality measures the extent to which a node lies on paths between other nodes by determining all the shortest paths (geodesic paths) between every pair of nodes and then counting how many times a node is on the shortest path between two others (Newman, 2010). If node *i* lies on the geodesic path from node *s* to node *t*, then the variable $n_{st}^{i}$ is 1, and it is 0 otherwise. The betweenness centrality *x*_*i*_ of node *i* is then given by:

(8) $$\begin{array}{r} x_{i}=\underset{st}{\sum }n_{st}^{i} \end{array}$$

## Results

Figure 1 shows the circuit of interactions of SARS-CoV-2 proteins with host processes, which was drawn from the experimental data available (Gordon et al., 2020; Wu et al., 2020). This circuit indicates that the virus principally alters the normal vesicular trafficking process of the host. Nine viral proteins (Nsp6, Nsp7, Nsp10, Nsp13, Nsp15, orf8, E, and M) modify the structure and function of the Endoplasmic Reticulum (ER) and Golgi Apparatus (GA) (Masters, 2006; McBride and Fielding, 2012; Steward, 2020). The presence of viral proteins also modifies other cellular processes like nuclear transport (Nsp 9, Nsp 15, and orf6), mitochondrial activity (Nsp4, Nsp8, and orf9c), cytoskeleton structure and function (Nsp1 and Nsp13), lipid synthesis and modification (Nsp2 and S), host DNA replication and regulation (Nsp1, Nsp5, Nsp8, and Nsp13), and host–virus RNA processing (Nsp2, Nsp8, and N) (Gordon et al., 2020). Figure 1 also points to the fact that Nsp8, Nsp13, N, orf6, and orf9b modify and take control of different signaling pathways. Orf6 produces alterations in signaling pathways that include activation of apoptosis via caspase-3-mediated, ER stress, and JNK-dependent pathways (Ye et al., 2008; McBride and Fielding, 2012). Orf6 is also a type I Interferon (IFN) antagonist, and its expression suppresses the induction of both IFN and the IFN signaling pathways (Frieman et al., 2007). N protein modifies the TGF-β signaling pathway to block apoptosis of infected host cells but can also induce apoptosis by activation of the mitochondrial pathway. This protein promotes NF-κB binding to the COX-2 promoter, causing inflammation of the lungs by activating COX-2 gene expression (Weiss and Leibowitz, 2011).

**Figure 2:**
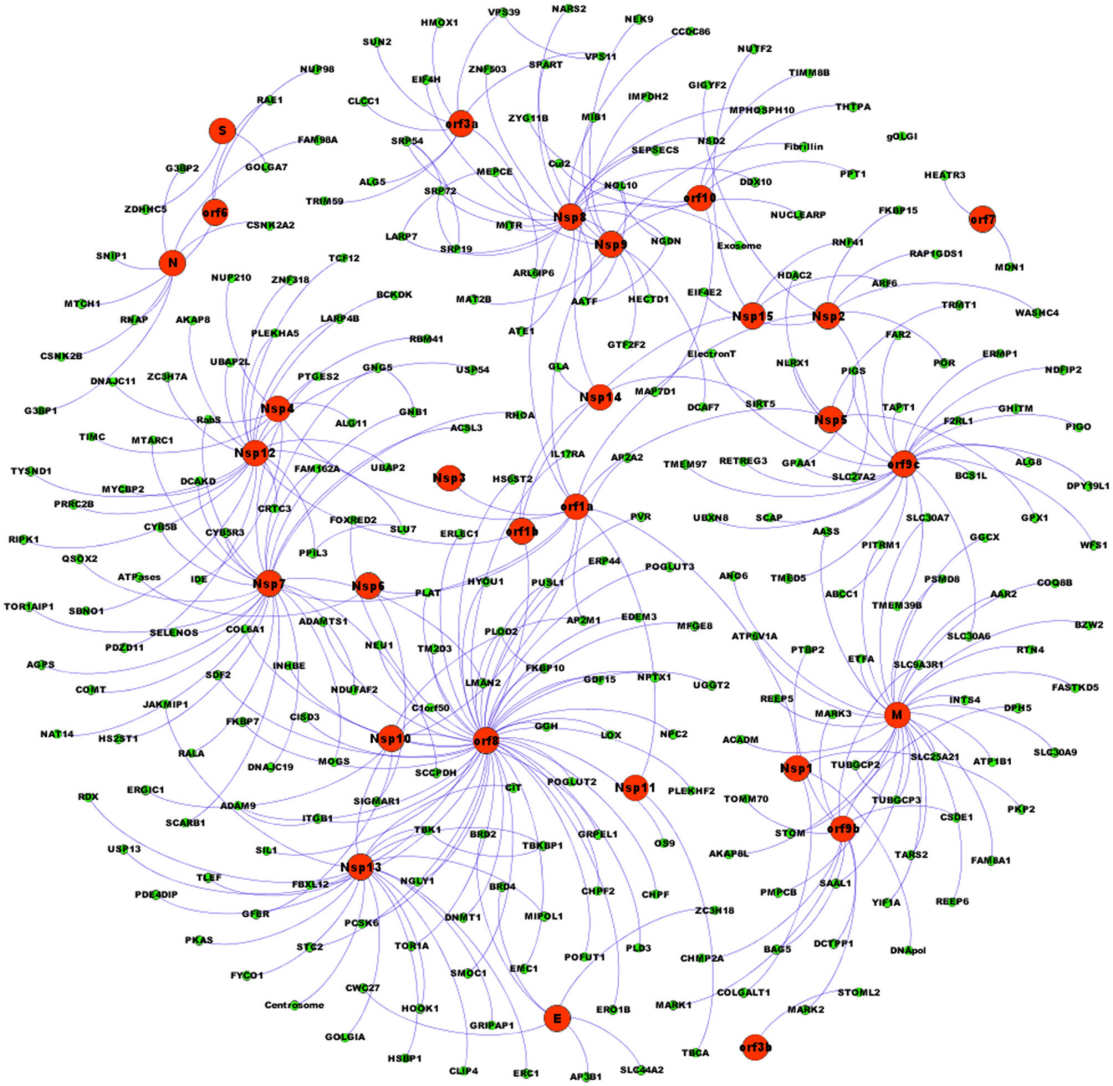
SARS-CoV-2 network. Network representation of the SARS-CoV-2 interactome was produced with the Fruchterman-Reingold algorithm. Red circles represent viral proteins. Green circles represent host proteins. Blue lines represent undirected links. The most connected viral protein is orf8.

Figure 2 shows the detailed SARS-CoV-2 undirected network representation made from the interactome obtained by Gordon et al. (2020), in which orf8, M, and Nsp7 are the most connected nodes. The network consists of 298 nodes, and Table 1 shows the basic statistics of its top 25 most connected nodes. The statistical properties of the complete network are presented in Table S1.

**Figure 3:**
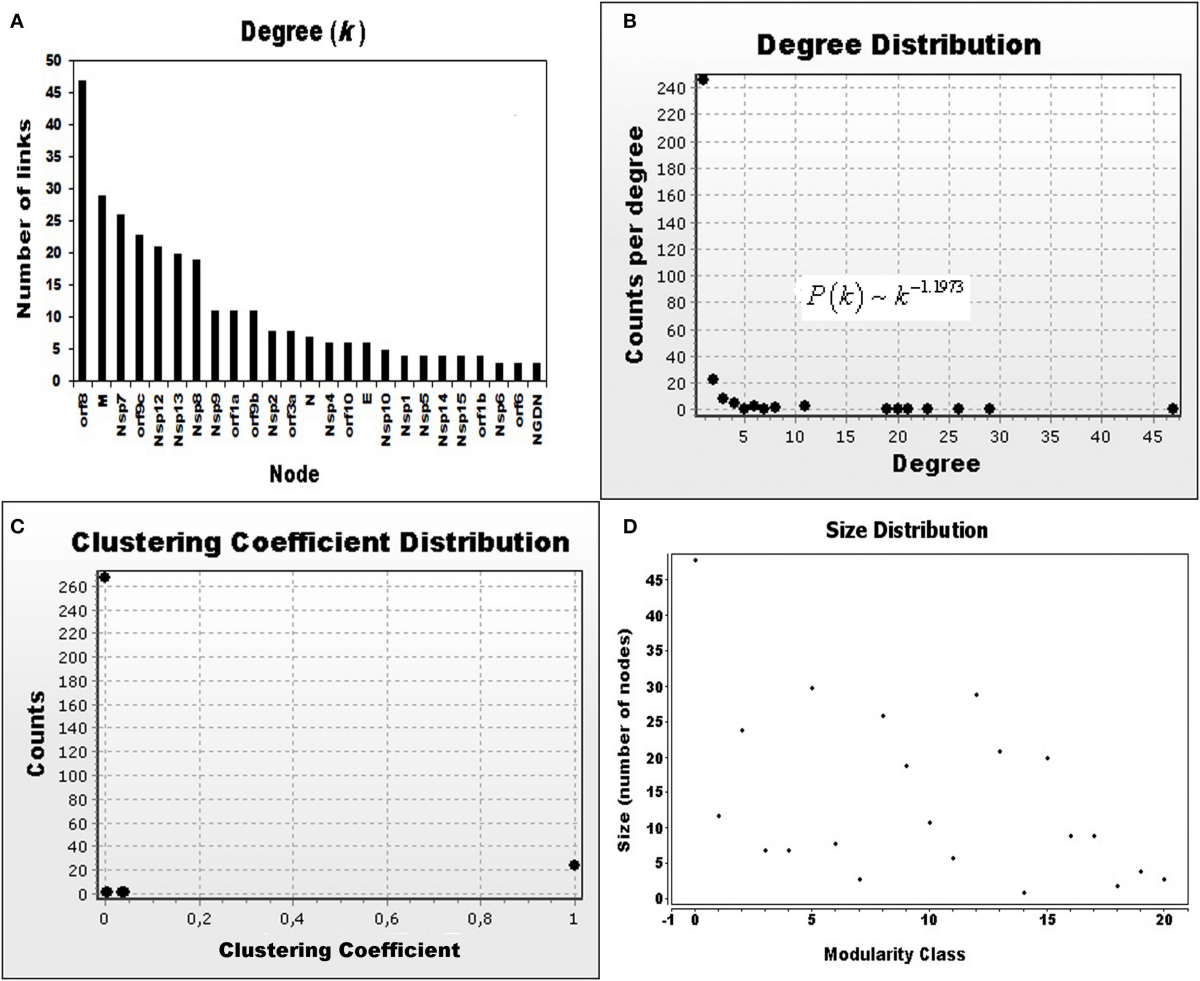
Basic statistical properties of the SARS-CoV-2 network. **(A)** Degrees of the 25 most connected nodes of the network. The orf8, M, and Nsp7 proteins have the largest numbers of links. The y-axis indicates the number of links per protein and the x-axis the proteins (nodes) of the network. **(B)** Degree distribution of the 298 nodes of the network. The degree distribution is a decaying exponential in which most of the proteins have only one link, and protein orf8 has 47 links. The distribution follows the power-law *P*(*k*) ~ *k*^−1.1973^. In this graph, the y-axis indicates the number of proteins that have the value of the degree indicated in the x-axis. **(C)** Clustering coefficient distribution of the network. The y-axis represents the number of nodes with the corresponding clustering coefficient value indicated in the x-axis. A high number of nodes have a clustering coefficient of 0, which indicates a poorly connected structure due to the presence of viral proteins. **(D)** Modularity of the network. The y-axis represents the number of nodes that belong to one of the modularity classes indicated in the x-axis. This graph suggests that the SARS-CoV-2 network is not random but has a hierarchical structure.

Figure 2, Table 1 and Table S2 show that most of the links are concentrated to only three virus proteins: orf8 with 47 links, M with 29 links, and Nsp7 with 26 links. The rest of the nodes have a degree value that decays in an exponential form, as shown in Figure 3A. Furthermore, Figure 3B clearly indicates that the degree distribution (Equation 1) is a decaying exponential, with 246 nodes with degree 1 and only six nodes with a degree above 20. This degree distribution obeys the power law $P\left(k\right)\tilde{\;}k^{-\gamma }$(Equation 2), in which the estimated value of the exponent is γ = 1.1973 (*R*-squared = 0.7148). The 95% confidence interval for the value of γ is 0.7368 < γ < 1.6308, which is lower than the value calculated for most scale-free biological networks (Voit, 2013) but high enough to assure that the SARS-CoV-2 network has a scale-free structure in which orf8, N, Nsp7, orf9c, Nsp12, and Nsp13 are the most connected nodes (hubs) of the net.

Table 1 shows the value of the clustering coefficient (Equation 3) for the top 25 nodes of the network, and Table S1 shows the values for all nodes. Figure 3C shows the clustering coefficient distribution for the complete network, in which the hubs orf8, M. Nsp7, orf9c, Nsp12, Nsp13, and Nsp8 have a small clustering coefficient, indicating that their neighbors are scarcely connected between them. Furthermore, 267 nodes have a clustering coefficient of zero, indicating that their neighbors have no links between them. The viral proteins Nsp9, orf1a, orf9b, Nsp2, N, Nsp4, orf10, E, Nsp10, Nsp1, Nsp5, Nsp14, Nsp15, orf1b, Nsp6, orf6, Nsp11, orf7, S, Nsp3, and orf3b belong to this group. None of the viral proteins has a clustering coefficient of 1. Finally, the average clustering coefficient of the network (Equation 4) is 0.463, which corresponds to a network with a small density of connections between the neighbors of each node.

The network has a modularity value of 0.85 (Equation 5), which is far from the negative value for a random network. Figure 3D shows that the network has 21 clusters with sizes that range from 1 to 49 modes in this example (Table 1 and Table S1), indicating that the number of nodes distributed between the 21 clusters is larger than the expected number due to randomness. Besides the original cellular proteins that belong to a high modularity class (Table S1), viral proteins orf8, M, orf9b, N, orf10, E, orf6, orf7, and S also belong to a high modularity class [see diagrams in Gordon et al. (2020)]. The values of closeness centrality (Equation 7) and betweenness centrality (Equation 8) presented in Table S1 suggest that SARS-CoV-2 is not a random network but has a hierarchical structure.

## Discussion

Every biological system can be represented as a network. A network is a simplified representation of reality that reduces a system to an abstract structure, capturing only the basics of connection patterns. A certain amount of information about the original system is usually lost in the process of reducing it to a network representation. However, the particular pattern of connections between components, i.e., the structure of the network, has a big effect on the behavior of the system (Newman, 2010). As a consequence, the knowledge of the structure of a network and is properties (degree, clustering degree distribution, etc.) is a tool for understanding the particular form of operation of a net.

Thus, the construction of the SARS-CoV-2 network, as a first approximation, involves the simplification (maybe the oversimplification) of some processes. Some nodes and links were not taken into consideration (the activity of M^prot^, the frame-shift that allows the expression of orf1b, and details of the vesicular trafficking process, among others) because the principal objective of this work is to determine the overall pattern of connections created by viral proteins during infection and to evaluate the basic statistical properties of the network. As a consequence, a second objective is to use this knowledge to determine which nodes of the network can be targets of therapeutic drugs (Steward, 2020).

With respect to the first objective, results obtained from the statistical analysis of the SARS-CoV-2 network (Figure 2, Table 1 and Table S1) indicate that the presence of the viral proteins in the host produces a new host–virus hybrid network (Figures 1, 2) in which the viral proteins interact with 273 host proteins in a pattern of connections with a modular hierarchical scale-free structure. Only six viral nodes influence the activity of 166 host nodes (59.7% of the total host nodes) in different cellular processes (Figures 1, 2, Table 1 and Table S1). In this set of six highly connected nodes, three of them predominate, with 102 links. Orf8 has 47 links; it is a glycoprotein that forms spikes at the surface of the SARS-CoV-2 virion envelope and participates in the fusion of viral and cellular membranes, leading to virus entry into the host cell (McBride and Fielding, 2012; Walls et al., 2020). One target of orf8 is the Tor1a (Torsin-1a) protein, whose function is related to the quality control of protein folding in the ER (Hill et al., 2018; Gordon et al., 2020). M protein has 29 links and is a homomultimer that synergizes with E protein in the budding compartment of the host cell, which is located between the ER and the GA. M protein also interacts with N protein in the packing of viral RNA into the virus (Masters, 2006; McBride and Fielding, 2012). Nsp7 has 26 links and forms a hexadecamer with Nsp8 (eight subunits of each). This hexadecamer acts as a primase in viral replication. A target of Nsp7 is COMT (Catechol O-methyltransferase) protein, which catalyzes the O-methylation, and thereby the inactivation, of catecholamine neurotransmitters and catechol hormones (Tai and Wu, 2002).

The low clustering coefficient of practically all nodes (Figure 3D and Table 1) indicates that viral infection produces a low-connectivity network structure. Experimental data that support this fact (Gordon et al., 2020) also show that viral proteins practically act independently one from the other because there are no direct links between them (Newman, 2010; Mengistu et al., 2016). They synergize their effects when converging in different cellular processes (Figure 1).

An important problem in network theory is to know when a particular network is random or has another type of structure. Both random and non-random networks can have an exponential degree distribution and low values of the clustering coefficient for each of their nodes. However, random networks have a negative modularity value, in contrast with the positive value (~ 0.85) calculated for the SARS-CoV-2 undirected network (Brandes et al., 2008). This result suggests that the virus does not alter the host network at random but reorganizes it into a hierarchical, efficient, and robust structure, in which each viral hub has the information to execute a particular function in the ensemble of the new virus. Figure 3D shows this tendency of viral proteins to form clusters (Gordon et al., 2020) in which orf8, M protein, and Nsp7 belong to clusters of high modularity class. This kind of network structure is highly resistant to the random deletion of a single node because the remaining ones stay active, i.e., the elimination of a particular node does not assure the destruction of the whole network (Mengistu et al., 2016). However, pharmacological attacks capable of deleting the more connected hubs can fracture the network and eliminate it (Cohen et al., 2000). The robustness of the SARS-CoV-2 network raises a question: how many hubs is it necessary to attack to effectively defeat COVID19? (Mengistu et al., 2016). At this moment, there is not an answer to this question. However, as a first approximation, this work proposes the hypothesis that a pharmacological attack on the more connected nodes (orf8, M protein, and Nsp7) will fracture the viral network, stopping the infection.

With respect to the second objective of this work, there are no therapeutic drugs that can directly attack these proteins, but there are FDA-approved drugs than can block the effect of these three hubs on downstream nodes. In particular, orf8 is a target of Rapamycin, which also targets Nsp2 and N protein. Rapamycin targets Tor1a, blocking its activity in the quality control of protein folding in the ER and disrupting the effects of orf8. Rapamycin could be a good candidate to block the activity of this hub, but this macrolide compound has strong immunosuppressant effects (Mannick et al., 2018). An alternative is FK-506, which is a macrolide lactone calcineurin inhibitor that also has immunosuppressant activity (Gordon et al., 2020). Other non-approved therapeutic drugs that could be alternatives for attacking orf8 are listed in the works of Gordon et al. (2020) and Steward (2020).

There are no reports of therapeutic drugs against the M protein hub, although Gordon et al. (2020) suggests that it is sensitive to Bafilomycin A1. This drug is a macrolide antibiotic that specifically targets the vacuolar-type H^+^ -ATPase (V-ATPase) enzyme, a membrane-spanning proton pump that acidifies either the extracellular environment or intracellular organelles.

The Nsp7 hub can be targeted by three drugs: a) Entacapone, which is a selective and reversible inhibitor of the enzyme COMT; b) Indomethacin, which is a cyclooxygenase inhibitor; c) Metformin, which is a biguanide antihyperglycemic agent that targets NDUFA12L protein, inducing a mild and transient inhibition of the mitochondrial respiratory chain complex I (Gordon et al., 2020; Steward, 2020).

Table 1 shows other FDA therapeutic agents that indirectly disrupt the functioning of SARS-CoV-2 network. Although orf1b is a poor connected node, with a low modularity class value, it is the target of the antiviral drug Remdesivir, which is a FDA-approved antiviral drug (Scavone et al., 2020). From a theoretical point of view, the combinations of drugs like *Rapamycin-Bafilomycin A1-Entacapone* can defeat the infection; however, the side effects of these drugs could be harmful for the patient. The combination *Rapamycin- Remdesivir-Chloroquine* could also be an alternative (Table 1). All of these suggestions need experimental verification and clinical evaluation. It is clear that more research is necessary to design drugs that can target the orf8, M, and Nsp7 proteins with fewer collateral effects.

This brief research report is an advance of the project “Dynamics of the SARS-CoV-2 network” and is based on the experimental data available at the moment. This report establishes the form in which the viral proteins restructure the host protein network giving rise to a modular hierarchical scale-free one, in which three viral nodes (orf8, M, and Nsp7) take control of a great number of cellular processes. A therapeutic attack on these hubs can increase the probability of defeating viral infection. More research is necessary to design drugs that directly target the orf8, M, and Nsp7 proteins as a compliment or an alternative to a vaccine.

## Data Availability Statement

All datasets presented in this study are included in the article/ Supplementary Material.

## Ethics Statement

Ethical review and approval was not required for the animal study because it is a theoretical work.

## Author Contributions

The author confirms being the sole contributor of this work and has approved it for publication.

## Conflict of Interest

The author declares that the research was conducted in the absence of any commercial or financial relationships that could be construed as a potential conflict of interest.
