# Supplementary material for: SARS-CoV-2 Molecular Network Structure
Source: Front Physiol. 2020 Jul 10;11:870. doi: 10.3389/fphys.2020.00870 (PMC7365879; doi:10.3389/fphys.2020.00870)
Supplement: Supplementary file 1 [file Table_1.docx]

Supplementary Material 1

**Table S1**. Statistical properties of the SARS-CoV-2 network.

| **Protein** | **Degree** | **Clustering Coefficient** | **Closeness Centrality** | **Betweenness Centrality** | **Modularity Class** |
| --- | --- | --- | --- | --- | --- |
| orf8 | 47 | 0.00185 | 1 | 1079 | 13 |
| M | 29 | 0.002463 | 1 | 405 | 19 |
| Nsp7 | 26 | 0.003077 | 0.430622 | 1924 | 3 |
| orf9c | 23 | 0.003953 | 1 | 252 | 15 |
| Nsp12 | 21 | 0.004762 | 0.480392 | 769 | 6 |
| Nsp13 | 20 | 0.005263 | 0.471154 | 740 | 8 |
| Nsp8 | 19 | 0.040936 | 0.403587 | 1442 | 4 |
| Nsp9 | 11 | 0 | 0.376569 | 845 | 0 |
| orf1a | 11 | 0 | 0.529412 | 3380 | 2 |
| orf9b | 11 | 0 | 1 | 55 | 14 |
| Nsp2 | 8 | 0 | 0.367347 | 602 | 2 |
| orf3a | 8 | 0.035714 | 1 | 27 | 9 |
| N | 7 | 0 | 1 | 21 | 18 |
| Nsp4 | 6 | 0 | 0.361446 | 435 | 2 |
| orf10 | 6 | 0 | 1 | 15 | 16 |
| E | 6 | 0 | 1 | 15 | 17 |
| Nsp10 | 5 | 0 | 0.361446 | 434 | 5 |
| Nsp1 | 4 | 0 | 0.355731 | 264 | 2 |
| Nsp5 | 4 | 0 | 0.355731 | 264 | 2 |
| Nsp14 | 4 | 0 | 0.360294 | 141 | 1 |
| Nsp15 | 4 | 0 | 0.360294 | 141 | 1 |
| orf1b | 4 | 0 | 0.521277 | 764 | 1 |
| Nsp6 | 3 | 0 | 0.352941 | 177 | 2 |
| orf6 | 3 | 0 | 1 | 3 | 11 |
| NGDN | 3 | 1 | 0.290323 | 0 | 4 |
| NOL10 | 3 | 1 | 0.290323 | 0 | 4 |
| AATF | 3 | 1 | 0.290323 | 0 | 4 |
| SRP54 | 3 | 1 | 0.290323 | 0 | 4 |
| SRP19 | 3 | 1 | 0.290323 | 0 | 4 |
| SRP72 | 3 | 1 | 0.290323 | 0 | 4 |
| Nsp11 | 2 | 0 | 0.350195 | 89 | 2 |
| orf7 | 2 | 0 | 1 | 1 | 12 |
| S | 2 | 0 | 1 | 1 | 20 |
| GNG5 | 2 | 1 | 0.30303 | 0 | 3 |
| GNB1 | 2 | 1 | 0.30303 | 0 | 3 |
| LARP7 | 2 | 1 | 0.289389 | 0 | 4 |
| MEPCE | 2 | 1 | 0.289389 | 0 | 4 |
| AP2M1 | 2 | 0 | 0.267857 | 89 | 5 |
| PPIL3 | 2 | 1 | 0.328859 | 0 | 6 |
| SLU7 | 2 | 1 | 0.328859 | 0 | 6 |
| TBKBP1 | 2 | 1 | 0.324503 | 0 | 8 |
| TBK1 | 2 | 1 | 0.324503 | 0 | 8 |
| VPS11 | 2 | 1 | 0.571429 | 0 | 9 |
| VPS39 | 2 | 1 | 0.571429 | 0 | 9 |
| ITGB1 | 2 | 1 | 0.51087 | 0 | 13 |
| ADAM9 | 2 | 1 | 0.51087 | 0 | 13 |
| FKBP10 | 2 | 1 | 0.51087 | 0 | 13 |
| PLOD2 | 2 | 1 | 0.51087 | 0 | 13 |
| PIGS | 2 | 1 | 0.522727 | 0 | 15 |
| GPAA1 | 2 | 1 | 0.522727 | 0 | 15 |
| TUBGCP3 | 2 | 1 | 0.517857 | 0 | 19 |
| TUBGCP2 | 2 | 1 | 0.517857 | 0 | 19 |
| Nsp3 | 1 | 0 | 0.34749 | 0 | 2 |
| orf3b | 1 | 0 | 1 | 0 | 10 |
| DNApol | 1 | 0 | 0.263158 | 0 | 2 |
| PKP2 | 1 | 0 | 0.263158 | 0 | 2 |
| COLGALT1 | 1 | 0 | 0.263158 | 0 | 2 |
| FKBP15 | 1 | 0 | 0.269461 | 0 | 2 |
| WASHC4 | 1 | 0 | 0.269461 | 0 | 2 |
| POR | 1 | 0 | 0.269461 | 0 | 2 |
| GIGYF2 | 1 | 0 | 0.269461 | 0 | 2 |
| EIF4E2 | 1 | 0 | 0.269461 | 0 | 2 |
| SLC27A2 | 1 | 0 | 0.269461 | 0 | 2 |
| RAP1GDS1 | 1 | 0 | 0.269461 | 0 | 2 |
| TIMC | 1 | 0 | 0.266272 | 0 | 2 |
| IDE | 1 | 0 | 0.266272 | 0 | 2 |
| DNAJC11 | 1 | 0 | 0.266272 | 0 | 2 |
| NUP210 | 1 | 0 | 0.266272 | 0 | 2 |
| ALG11 | 1 | 0 | 0.266272 | 0 | 2 |
| HDAC2 | 1 | 0 | 0.263158 | 0 | 2 |
| TRMT1 | 1 | 0 | 0.263158 | 0 | 2 |
| GPX1 | 1 | 0 | 0.263158 | 0 | 2 |
| ATPases | 1 | 0 | 0.261628 | 0 | 2 |
| SIGMAR1 | 1 | 0 | 0.261628 | 0 | 2 |
| RabS | 1 | 0 | 0.302013 | 0 | 3 |
| LMAN2 | 1 | 0 | 0.302013 | 0 | 3 |
| NDUFAF2 | 1 | 0 | 0.302013 | 0 | 3 |
| COMT | 1 | 0 | 0.302013 | 0 | 3 |
| HS2ST1 | 1 | 0 | 0.302013 | 0 | 3 |
| QSOX2 | 1 | 0 | 0.302013 | 0 | 3 |
| RALA | 1 | 0 | 0.302013 | 0 | 3 |
| RHOA | 1 | 0 | 0.302013 | 0 | 3 |
| MTARC1 | 1 | 0 | 0.302013 | 0 | 3 |
| SCCPDH | 1 | 0 | 0.302013 | 0 | 3 |
| SELENOS | 1 | 0 | 0.302013 | 0 | 3 |
| AGPS | 1 | 0 | 0.302013 | 0 | 3 |
| TOR1AIP1 | 1 | 0 | 0.302013 | 0 | 3 |
| DNAJC19 | 1 | 0 | 0.302013 | 0 | 3 |
| MOGS | 1 | 0 | 0.302013 | 0 | 3 |
| ACSL3 | 1 | 0 | 0.302013 | 0 | 3 |
| FAM162A | 1 | 0 | 0.302013 | 0 | 3 |
| DCAKD | 1 | 0 | 0.302013 | 0 | 3 |
| NAT14 | 1 | 0 | 0.302013 | 0 | 3 |
| SCARB1 | 1 | 0 | 0.302013 | 0 | 3 |
| CYB5B | 1 | 0 | 0.302013 | 0 | 3 |
| PTGES2 | 1 | 0 | 0.302013 | 0 | 3 |
| CYB5R3 | 1 | 0 | 0.302013 | 0 | 3 |
| Exosome | 1 | 0 | 0.288462 | 0 | 4 |
| MITR | 1 | 0 | 0.288462 | 0 | 4 |
| SEPSECS | 1 | 0 | 0.288462 | 0 | 4 |
| ATE1 | 1 | 0 | 0.288462 | 0 | 4 |
| MPHOSPH10 | 1 | 0 | 0.288462 | 0 | 4 |
| NSD2 | 1 | 0 | 0.288462 | 0 | 4 |
| DDX10 | 1 | 0 | 0.288462 | 0 | 4 |
| HECTD1 | 1 | 0 | 0.288462 | 0 | 4 |
| CCDC86 | 1 | 0 | 0.288462 | 0 | 4 |
| NARS2 | 1 | 0 | 0.288462 | 0 | 4 |
| NUCLEARP | 1 | 0 | 0.27439 | 0 | 0 |
| MAT2B | 1 | 0 | 0.27439 | 0 | 0 |
| ZNF503 | 1 | 0 | 0.27439 | 0 | 0 |
| Fibrillin | 1 | 0 | 0.27439 | 0 | 0 |
| SPART | 1 | 0 | 0.27439 | 0 | 0 |
| MIB1 | 1 | 0 | 0.27439 | 0 | 0 |
| NEK9 | 1 | 0 | 0.27439 | 0 | 0 |
| GTF2F2 | 1 | 0 | 0.27439 | 0 | 0 |
| DCAF7 | 1 | 0 | 0.27439 | 0 | 0 |
| EIF4H | 1 | 0 | 0.27439 | 0 | 0 |
| GRPEL1 | 1 | 0 | 0.266272 | 0 | 5 |
| AP2A2 | 1 | 0 | 0.211765 | 0 | 5 |
| ERGIC1 | 1 | 0 | 0.266272 | 0 | 5 |
| GFER | 1 | 0 | 0.266272 | 0 | 5 |
| TBCA | 1 | 0 | 0.260116 | 0 | 2 |
| RIPK1 | 1 | 0 | 0.326667 | 0 | 6 |
| RBM41 | 1 | 0 | 0.326667 | 0 | 6 |
| PRRC2B | 1 | 0 | 0.326667 | 0 | 6 |
| PLEKHA5 | 1 | 0 | 0.326667 | 0 | 6 |
| PDZD11 | 1 | 0 | 0.326667 | 0 | 6 |
| MYCBP2 | 1 | 0 | 0.326667 | 0 | 6 |
| LARP4B | 1 | 0 | 0.326667 | 0 | 6 |
| CRTC3 | 1 | 0 | 0.326667 | 0 | 6 |
| AKAP8 | 1 | 0 | 0.326667 | 0 | 6 |
| UBAP2L | 1 | 0 | 0.326667 | 0 | 6 |
| UBAP2 | 1 | 0 | 0.326667 | 0 | 6 |
| ZNF318 | 1 | 0 | 0.326667 | 0 | 6 |
| ZC3H7A | 1 | 0 | 0.326667 | 0 | 6 |
| BCKDK | 1 | 0 | 0.326667 | 0 | 6 |
| USP54 | 1 | 0 | 0.326667 | 0 | 6 |
| TYSND1 | 1 | 0 | 0.326667 | 0 | 6 |
| TCF12 | 1 | 0 | 0.326667 | 0 | 6 |
| SBNO1 | 1 | 0 | 0.326667 | 0 | 6 |
| Centrosome | 1 | 0 | 0.322368 | 0 | 8 |
| PKAS | 1 | 0 | 0.322368 | 0 | 8 |
| PDE4DIP | 1 | 0 | 0.322368 | 0 | 8 |
| HOOK1 | 1 | 0 | 0.322368 | 0 | 8 |
| GOLGIA | 1 | 0 | 0.322368 | 0 | 8 |
| FYCO1 | 1 | 0 | 0.322368 | 0 | 8 |
| HSBP1 | 1 | 0 | 0.322368 | 0 | 8 |
| USP13 | 1 | 0 | 0.322368 | 0 | 8 |
| MIPOL1 | 1 | 0 | 0.322368 | 0 | 8 |
| TLEF | 1 | 0 | 0.322368 | 0 | 8 |
| CIT | 1 | 0 | 0.322368 | 0 | 8 |
| GRIPAP1 | 1 | 0 | 0.322368 | 0 | 8 |
| JAKMIP1 | 1 | 0 | 0.322368 | 0 | 8 |
| CLIP4 | 1 | 0 | 0.322368 | 0 | 8 |
| RDX | 1 | 0 | 0.322368 | 0 | 8 |
| C1orf50 | 1 | 0 | 0.322368 | 0 | 8 |
| ERC1 | 1 | 0 | 0.322368 | 0 | 8 |
| GLA | 1 | 0 | 0.266304 | 0 | 1 |
| SIRT5 | 1 | 0 | 0.266304 | 0 | 1 |
| IMPDH2 | 1 | 0 | 0.266304 | 0 | 1 |
| ARF6 | 1 | 0 | 0.266304 | 0 | 1 |
| NUTF2 | 1 | 0 | 0.266304 | 0 | 1 |
| RNF41 | 1 | 0 | 0.266304 | 0 | 1 |
| SUN2 | 1 | 0 | 0.533333 | 0 | 9 |
| CLCC1 | 1 | 0 | 0.533333 | 0 | 9 |
| ARL6IP6 | 1 | 0 | 0.533333 | 0 | 9 |
| ALG5 | 1 | 0 | 0.533333 | 0 | 9 |
| TRIM59 | 1 | 0 | 0.533333 | 0 | 9 |
| HMOX1 | 1 | 0 | 0.533333 | 0 | 9 |
| STOML2 | 1 | 0 | 1 | 0 | 10 |
| NUP98 | 1 | 0 | 0.6 | 0 | 11 |
| RAE1 | 1 | 0 | 0.6 | 0 | 11 |
| MTCH1 | 1 | 0 | 0.6 | 0 | 11 |
| HEATR3 | 1 | 0 | 0.666667 | 0 | 12 |
| MDN1 | 1 | 0 | 0.666667 | 0 | 12 |
| NEU1 | 1 | 0 | 0.505376 | 0 | 13 |
| PLAT | 1 | 0 | 0.505376 | 0 | 13 |
| POGLUT2 | 1 | 0 | 0.505376 | 0 | 13 |
| STC2 | 1 | 0 | 0.505376 | 0 | 13 |
| NPTX1 | 1 | 0 | 0.505376 | 0 | 13 |
| POFUT1 | 1 | 0 | 0.505376 | 0 | 13 |
| HS6ST2 | 1 | 0 | 0.505376 | 0 | 13 |
| MFGE8 | 1 | 0 | 0.505376 | 0 | 13 |
| ERP44 | 1 | 0 | 0.505376 | 0 | 13 |
| CHPF2 | 1 | 0 | 0.505376 | 0 | 13 |
| EMC1 | 1 | 0 | 0.505376 | 0 | 13 |
| DNMT1 | 1 | 0 | 0.505376 | 0 | 13 |
| PCSK6 | 1 | 0 | 0.505376 | 0 | 13 |
| PLD3 | 1 | 0 | 0.505376 | 0 | 13 |
| PUSL1 | 1 | 0 | 0.505376 | 0 | 13 |
| IL17RA | 1 | 0 | 0.505376 | 0 | 13 |
| GGH | 1 | 0 | 0.505376 | 0 | 13 |
| FKBP7 | 1 | 0 | 0.505376 | 0 | 13 |
| COL6A1 | 1 | 0 | 0.505376 | 0 | 13 |
| PVR | 1 | 0 | 0.505376 | 0 | 13 |
| LOX | 1 | 0 | 0.505376 | 0 | 13 |
| CHPF | 1 | 0 | 0.505376 | 0 | 13 |
| NPC2 | 1 | 0 | 0.505376 | 0 | 13 |
| TM2D3 | 1 | 0 | 0.505376 | 0 | 13 |
| ADAMTS1 | 1 | 0 | 0.505376 | 0 | 13 |
| SDF2 | 1 | 0 | 0.505376 | 0 | 13 |
| FOXRED2 | 1 | 0 | 0.505376 | 0 | 13 |
| TOR1A | 1 | 0 | 0.505376 | 0 | 13 |
| NGLY1 | 1 | 0 | 0.505376 | 0 | 13 |
| HYOU1 | 1 | 0 | 0.505376 | 0 | 13 |
| SIL1 | 1 | 0 | 0.505376 | 0 | 13 |
| ERO1B | 1 | 0 | 0.505376 | 0 | 13 |
| UGGT2 | 1 | 0 | 0.505376 | 0 | 13 |
| OS9 | 1 | 0 | 0.505376 | 0 | 13 |
| ERLEC1 | 1 | 0 | 0.505376 | 0 | 13 |
| EDEM3 | 1 | 0 | 0.505376 | 0 | 13 |
| FBXL12 | 1 | 0 | 0.505376 | 0 | 13 |
| POGLUT3 | 1 | 0 | 0.505376 | 0 | 13 |
| PLEKHF2 | 1 | 0 | 0.505376 | 0 | 13 |
| CISD3 | 1 | 0 | 0.505376 | 0 | 13 |
| INHBE | 1 | 0 | 0.505376 | 0 | 13 |
| GDF15 | 1 | 0 | 0.505376 | 0 | 13 |
| SMOC1 | 1 | 0 | 0.505376 | 0 | 13 |
| MARK3 | 1 | 0 | 0.52381 | 0 | 14 |
| MARK1 | 1 | 0 | 0.52381 | 0 | 14 |
| MARK2 | 1 | 0 | 0.52381 | 0 | 14 |
| BAG5 | 1 | 0 | 0.52381 | 0 | 14 |
| PTBP2 | 1 | 0 | 0.52381 | 0 | 14 |
| CSDE1 | 1 | 0 | 0.52381 | 0 | 14 |
| DPH5 | 1 | 0 | 0.52381 | 0 | 14 |
| CHMP2A | 1 | 0 | 0.52381 | 0 | 14 |
| SLC9A3R1 | 1 | 0 | 0.52381 | 0 | 14 |
| TOMM70 | 1 | 0 | 0.52381 | 0 | 14 |
| DCTPP1 | 1 | 0 | 0.52381 | 0 | 14 |
| ElectronT | 1 | 0 | 0.511111 | 0 | 15 |
| FAR2 | 1 | 0 | 0.511111 | 0 | 15 |
| WFS1 | 1 | 0 | 0.511111 | 0 | 15 |
| PIGO | 1 | 0 | 0.511111 | 0 | 15 |
| RETREG3 | 1 | 0 | 0.511111 | 0 | 15 |
| UBXN8 | 1 | 0 | 0.511111 | 0 | 15 |
| NLRX1 | 1 | 0 | 0.511111 | 0 | 15 |
| TMEM97 | 1 | 0 | 0.511111 | 0 | 15 |
| ERMP1 | 1 | 0 | 0.511111 | 0 | 15 |
| TAPT1 | 1 | 0 | 0.511111 | 0 | 15 |
| SLC30A6 | 1 | 0 | 0.511111 | 0 | 15 |
| TMED5 | 1 | 0 | 0.511111 | 0 | 15 |
| SCAP | 1 | 0 | 0.511111 | 0 | 15 |
| BCS1L | 1 | 0 | 0.511111 | 0 | 15 |
| NDFIP2 | 1 | 0 | 0.511111 | 0 | 15 |
| DPY19L1 | 1 | 0 | 0.511111 | 0 | 15 |
| F2RL1 | 1 | 0 | 0.511111 | 0 | 15 |
| GHITM | 1 | 0 | 0.511111 | 0 | 15 |
| ABCC1 | 1 | 0 | 0.511111 | 0 | 15 |
| TMEM39B | 1 | 0 | 0.511111 | 0 | 15 |
| ALG8 | 1 | 0 | 0.511111 | 0 | 15 |
| Cul2 | 1 | 0 | 0.545455 | 0 | 16 |
| ZYG11B | 1 | 0 | 0.545455 | 0 | 16 |
| PPT1 | 1 | 0 | 0.545455 | 0 | 16 |
| TIMM8B | 1 | 0 | 0.545455 | 0 | 16 |
| THTPA | 1 | 0 | 0.545455 | 0 | 16 |
| MAP7D1 | 1 | 0 | 0.545455 | 0 | 16 |
| BRD2 | 1 | 0 | 0.545455 | 0 | 17 |
| BRD4 | 1 | 0 | 0.545455 | 0 | 17 |
| SLC44A2 | 1 | 0 | 0.545455 | 0 | 17 |
| ZC3H18 | 1 | 0 | 0.545455 | 0 | 17 |
| AP3B1 | 1 | 0 | 0.545455 | 0 | 17 |
| CWC27 | 1 | 0 | 0.545455 | 0 | 17 |
| RNAP | 1 | 0 | 0.538462 | 0 | 18 |
| G3BP1 | 1 | 0 | 0.538462 | 0 | 18 |
| G3BP2 | 1 | 0 | 0.538462 | 0 | 18 |
| CSNK2A2 | 1 | 0 | 0.538462 | 0 | 18 |
| CSNK2B | 1 | 0 | 0.538462 | 0 | 18 |
| SNIP1 | 1 | 0 | 0.538462 | 0 | 18 |
| FAM98A | 1 | 0 | 0.538462 | 0 | 18 |
| PITRM1 | 1 | 0 | 0.508772 | 0 | 19 |
| INTS4 | 1 | 0 | 0.508772 | 0 | 19 |
| GGCX | 1 | 0 | 0.508772 | 0 | 19 |
| FASTKD5 | 1 | 0 | 0.508772 | 0 | 19 |
| FAM8A1 | 1 | 0 | 0.508772 | 0 | 19 |
| ETFA | 1 | 0 | 0.508772 | 0 | 19 |
| COQ8B | 1 | 0 | 0.508772 | 0 | 19 |
| BZW2 | 1 | 0 | 0.508772 | 0 | 19 |
| ATP6V1A | 1 | 0 | 0.508772 | 0 | 19 |
| ATP1B1 | 1 | 0 | 0.508772 | 0 | 19 |
| AASS | 1 | 0 | 0.508772 | 0 | 19 |
| ACADM | 1 | 0 | 0.508772 | 0 | 19 |
| AKAP8L | 1 | 0 | 0.508772 | 0 | 19 |
| ANO6 | 1 | 0 | 0.508772 | 0 | 19 |
| RTN4 | 1 | 0 | 0.508772 | 0 | 19 |
| YIF1A | 1 | 0 | 0.508772 | 0 | 19 |
| REEP5 | 1 | 0 | 0.508772 | 0 | 19 |
| REEP6 | 1 | 0 | 0.508772 | 0 | 19 |
| SLC30A9 | 1 | 0 | 0.508772 | 0 | 19 |
| SLC30A7 | 1 | 0 | 0.508772 | 0 | 19 |
| SLC25A21 | 1 | 0 | 0.508772 | 0 | 19 |
| SAAL1 | 1 | 0 | 0.508772 | 0 | 19 |
| AAR2 | 1 | 0 | 0.508772 | 0 | 19 |
| TARS2 | 1 | 0 | 0.508772 | 0 | 19 |
| STOM | 1 | 0 | 0.508772 | 0 | 19 |
| PSMD8 | 1 | 0 | 0.508772 | 0 | 19 |
| PMPCB | 1 | 0 | 0.508772 | 0 | 19 |
| GOLGA7 | 1 | 0 | 0.666667 | 0 | 20 |
| ZDHHC5 | 1 | 0 | 0.666667 | 0 | 20 |
