# Supplementary material for: SARS-CoV-2 Molecular Network Structure
Source: Front Physiol. 2020 Jul 10;11:870. doi: 10.3389/fphys.2020.00870 (PMC7365879; doi:10.3389/fphys.2020.00870)
Supplement: Supplementary file 2 [file Table_2.docx]

Supplementary Material 2

# Supplementary Table

**SM Table II**. – Protein-protein links of SARS-CoV-2 network.

| **Nodes** | **Protein** | **Id** | **Links** | **Source** | **Target** |
| --- | --- | --- | --- | --- | --- |
|  | Nsp1 | 0 |  | 0 | 29 |
|  | Nsp2 | 1 |  | 0 | 30 |
|  | Nsp3 | 2 |  | 0 | 31 |
|  | Nsp4 | 3 |  | 1 | 32 |
|  | Nsp5 | 4 |  | 1 | 33 |
|  | Nsp6 | 5 |  | 1 | 34 |
|  | Nsp7 | 6 |  | 1 | 35 |
|  | Nsp8 | 7 |  | 1 | 36 |
|  | Nsp9 | 8 |  | 1 | 37 |
|  | Nsp10 | 9 |  | 1 | 38 |
|  | Nsp11 | 10 |  | 3 | 39 |
|  | Nsp12 | 11 |  | 3 | 40 |
|  | Nsp13 | 12 |  | 3 | 41 |
|  | Nsp14 | 13 |  | 3 | 42 |
|  | Nsp15 | 14 |  | 3 | 43 |
|  | orf1a | 16 |  | 4 | 44 |
|  | orf1b | 17 |  | 4 | 45 |
|  | orf3a | 18 |  | 4 | 46 |
|  | orf3b | 19 |  | 5 | 47 |
|  | orf6 | 20 |  | 5 | 48 |
|  | orf7 | 21 |  | 6 | 49 |
|  | orf8 | 22 |  | 6 | 50 |
|  | orf9b | 23 |  | 6 | 51 |
|  | orf9c | 24 |  | 6 | 52 |
|  | orf10 | 25 |  | 51 | 52 |
|  | E | 26 |  | 6 | 53 |
|  | M | 27 |  | 6 | 54 |
|  | N | 28 |  | 6 | 55 |
|  | S | 29 |  | 6 | 56 |
|  | DNApol | 30 |  | 6 | 57 |
|  | PKP2 | 31 |  | 6 | 58 |
|  | COLGALT1 | 32 |  | 6 | 59 |
|  | FKBP15 | 33 |  | 6 | 60 |
|  | WASHC4 | 34 |  | 6 | 61 |
|  | POR | 35 |  | 6 | 62 |
|  | GIGYF2 | 36 |  | 6 | 63 |
|  | EIF4E2 | 37 |  | 6 | 64 |
|  | SLC27A2 | 38 |  | 6 | 65 |
|  | RAP1GDS1 | 39 |  | 6 | 66 |
|  | TIMC | 40 |  | 6 | 67 |
|  | IDE | 41 |  | 6 | 68 |
|  | DNAJC11 | 42 |  | 6 | 69 |
|  | NUP210 | 43 |  | 6 | 70 |
|  | ALG11 | 44 |  | 6 | 71 |
|  | HDAC2 | 45 |  | 6 | 72 |
|  | TRMT1 | 46 |  | 6 | 73 |
|  | GPX1 | 47 |  | 7 | 74 |
|  | ATPases | 48 |  | 7 | 75 |
|  | SIGMAR1 | 49 |  | 7 | 76 |
|  | RabS | 50 |  | 75 | 76 |
|  | LMAN2 | 51 |  | 7 | 77 |
|  | GNG5 | 52 |  | 7 | 78 |
|  | GNB1 | 53 |  | 7 | 79 |
|  | NDUFAF2 | 54 |  | 7 | 80 |
|  | COMT | 55 |  | 7 | 81 |
|  | HS2ST1 | 56 |  | 7 | 82 |
|  | QSOX2 | 57 |  | 7 | 83 |
|  | RALA | 58 |  | 7 | 84 |
|  | RHOA | 59 |  | 7 | 85 |
|  | MTARC1 | 60 |  | 7 | 86 |
|  | SCCPDH | 61 |  | 84 | 85 |
|  | SELENOS | 62 |  | 84 | 86 |
|  | AGPS | 63 |  | 85 | 86 |
|  | TOR1AIP1 | 64 |  | 7 | 87 |
|  | DNAJC19 | 65 |  | 7 | 88 |
|  | MOGS | 66 |  | 7 | 89 |
|  | ACSL3 | 67 |  | 87 | 88 |
|  | FAM162A | 68 |  | 87 | 89 |
|  | DCAKD | 69 |  | 88 | 89 |
|  | NAT14 | 70 |  | 7 | 90 |
|  | SCARB1 | 71 |  | 7 | 91 |
|  | CYB5B | 72 |  | 8 | 92 |
|  | PTGES2 | 73 |  | 8 | 93 |
|  | CYB5R3 | 74 |  | 8 | 94 |
|  | Exosome | 75 |  | 8 | 95 |
|  | LARP7 | 76 |  | 8 | 96 |
|  | MEPCE | 77 |  | 8 | 97 |
|  | MITR | 78 |  | 8 | 98 |
|  | SEPSECS | 79 |  | 8 | 99 |
|  | ATE1 | 80 |  | 8 | 100 |
|  | MPHOSPH10 | 81 |  | 8 | 101 |
|  | NSD2 | 82 |  | 9 | 102 |
|  | DDX10 | 83 |  | 9 | 103 |
|  | HECTD1 | 84 |  | 103 | 104 |
|  | NGDN | 85 |  | 9 | 105 |
|  | NOL10 | 86 |  | 9 | 106 |
|  | AATF | 87 |  | 10 | 107 |
|  | SRP54 | 89 |  | 11 | 108 |
|  | SRP19 | 90 |  | 11 | 109 |
|  | SRP72 | 91 |  | 11 | 110 |
|  | CCDC86 | 92 |  | 11 | 111 |
|  | NARS2 | 93 |  | 11 | 112 |
|  | NUCLEARP | 94 |  | 11 | 113 |
|  | MAT2B | 95 |  | 11 | 114 |
|  | ZNF503 | 96 |  | 11 | 115 |
|  | Fibrillin | 97 |  | 11 | 116 |
|  | SPART | 98 |  | 11 | 117 |
|  | MIB1 | 99 |  | 11 | 118 |
|  | NEK9 | 100 |  | 11 | 119 |
|  | GTF2F2 | 101 |  | 11 | 120 |
|  | DCAF7 | 102 |  | 11 | 121 |
|  | EIF4H | 103 |  | 11 | 122 |
|  | GRPEL1 | 104 |  | 11 | 123 |
|  | AP2M1 | 105 |  | 11 | 124 |
|  | AP2A2 | 106 |  | 11 | 125 |
|  | ERGIC1 | 107 |  | 11 | 126 |
|  | GFER | 108 |  | 11 | 127 |
|  | TBCA | 109 |  | 126 | 127 |
|  | RIPK1 | 110 |  | 12 | 128 |
|  | RBM41 | 111 |  | 12 | 129 |
|  | PRRC2B | 112 |  | 12 | 130 |
|  | PLEKHA5 | 113 |  | 12 | 131 |
|  | PDZD11 | 114 |  | 12 | 133 |
|  | MYCBP2 | 115 |  | 12 | 134 |
|  | LARP4B | 116 |  | 12 | 135 |
|  | CRTC3 | 117 |  | 12 | 136 |
|  | AKAP8 | 118 |  | 12 | 137 |
|  | UBAP2L | 119 |  | 12 | 138 |
|  | UBAP2 | 120 |  | 12 | 139 |
|  | ZNF318 | 121 |  | 12 | 140 |
|  | ZC3H7A | 122 |  | 12 | 141 |
|  | BCKDK | 123 |  | 140 | 141 |
|  | USP54 | 124 |  | 12 | 142 |
|  | TYSND1 | 125 |  | 12 | 143 |
|  | TCF12 | 126 |  | 12 | 144 |
|  | SBNO1 | 127 |  | 12 | 145 |
|  | PPIL3 | 128 |  | 12 | 146 |
|  | SLU7 | 129 |  | 12 | 147 |
|  | Centrosome | 130 |  | 13 | 148 |
|  | PKAS | 131 |  | 13 | 149 |
|  | PDE4DIP | 132 |  | 13 | 150 |
|  | HOOK1 | 133 |  | 14 | 151 |
|  | gOLGI | 134 |  | 14 | 152 |
|  | GOLGIA | 135 |  | 14 | 153 |
|  | FYCO1 | 136 |  | 15 | 0 |
|  | HSBP1 | 137 |  | 15 | 1 |
|  | USP13 | 138 |  | 15 | 2 |
|  | MIPOL1 | 139 |  | 15 | 3 |
|  | TLEF | 140 |  | 15 | 4 |
|  | CIT | 141 |  | 15 | 5 |
|  | TBKBP1 | 142 |  | 15 | 6 |
|  | TBK1 | 143 |  | 15 | 7 |
|  | GRIPAP1 | 144 |  | 15 | 8 |
|  | JAKMIP1 | 145 |  | 15 | 9 |
|  | CLIP4 | 146 |  | 15 | 10 |
|  | RDX | 147 |  | 16 | 11 |
|  | C1orf50 | 148 |  | 16 | 12 |
|  | ERC1 | 149 |  | 16 | 13 |
|  | GLA | 150 |  | 16 | 14 |
|  | SIRT5 | 151 |  | 17 | 154 |
|  | IMPDH2 | 152 |  | 17 | 155 |
|  | ARF6 | 153 |  | 154 | 155 |
|  | NUTF2 | 154 |  | 17 | 156 |
|  | RNF41 | 155 |  | 17 | 157 |
|  | VPS11 | 156 |  | 17 | 158 |
|  | VPS39 | 157 |  | 17 | 159 |
|  | SUN2 | 158 |  | 17 | 160 |
|  | CLCC1 | 159 |  | 17 | 161 |
|  | ARL6IP6 | 160 |  | 18 | 162 |
|  | ALG5 | 161 |  | 19 | 163 |
|  | TRIM59 | 162 |  | 19 | 164 |
|  | HMOX1 | 163 |  | 19 | 165 |
|  | STOML2 | 164 |  | 20 | 166 |
|  | NUP98 | 165 |  | 20 | 167 |
|  | RAE1 | 166 |  | 21 | 168 |
|  | MTCH1 | 167 |  | 21 | 169 |
|  | HEATR3 | 168 |  | 21 | 170 |
|  | MDN1 | 169 |  | 21 | 171 |
|  | NEU1 | 170 |  | 21 | 172 |
|  | PLAT | 171 |  | 21 | 173 |
|  | POGLUT2 | 172 |  | 21 | 174 |
|  | STC2 | 173 |  | 21 | 175 |
|  | NPTX1 | 174 |  | 21 | 176 |
|  | POFUT1 | 175 |  | 21 | 177 |
|  | HS6ST2 | 176 |  | 21 | 178 |
|  | MFGE8 | 177 |  | 21 | 179 |
|  | ERP44 | 178 |  | 21 | 180 |
|  | CHPF2 | 179 |  | 21 | 181 |
|  | EMC1 | 180 |  | 180 | 181 |
|  | DNMT1 | 181 |  | 21 | 182 |
|  | ITGB1 | 182 |  | 21 | 183 |
|  | ADAM9 | 183 |  | 21 | 184 |
|  | PCSK6 | 184 |  | 21 | 185 |
|  | PLD3 | 185 |  | 21 | 186 |
|  | PUSL1 | 186 |  | 21 | 187 |
|  | IL17RA | 187 |  | 21 | 188 |
|  | GGH | 188 |  | 21 | 189 |
|  | FKBP7 | 189 |  | 188 | 189 |
|  | FKBP10 | 190 |  | 21 | 190 |
|  | PLOD2 | 191 |  | 21 | 191 |
|  | COL6A1 | 192 |  | 21 | 192 |
|  | PVR | 193 |  | 21 | 193 |
|  | LOX | 194 |  | 21 | 194 |
|  | CHPF | 195 |  | 21 | 195 |
|  | NPC2 | 196 |  | 21 | 196 |
|  | TM2D3 | 197 |  | 21 | 197 |
|  | ADAMTS1 | 198 |  | 21 | 198 |
|  | SDF2 | 199 |  | 21 | 199 |
|  | FOXRED2 | 200 |  | 21 | 200 |
|  | TOR1A | 201 |  | 21 | 201 |
|  | NGLY1 | 202 |  | 21 | 202 |
|  | HYOU1 | 203 |  | 21 | 203 |
|  | SIL1 | 204 |  | 21 | 204 |
|  | ERO1B | 205 |  | 21 | 205 |
|  | UGGT2 | 206 |  | 21 | 206 |
|  | OS9 | 207 |  | 21 | 207 |
|  | ERLEC1 | 208 |  | 21 | 208 |
|  | EDEM3 | 209 |  | 21 | 209 |
|  | FBXL12 | 210 |  | 21 | 210 |
|  | POGLUT3 | 211 |  | 21 | 211 |
|  | PLEKHF2 | 212 |  | 21 | 212 |
|  | CISD3 | 213 |  | 21 | 213 |
|  | INHBE | 214 |  | 21 | 214 |
|  | GDF15 | 215 |  | 22 | 215 |
|  | SMOC1 | 216 |  | 22 | 216 |
|  | MARK3 | 217 |  | 22 | 217 |
|  | MARK1 | 218 |  | 22 | 218 |
|  | MARK2 | 219 |  | 22 | 219 |
|  | BAG5 | 220 |  | 22 | 220 |
|  | PTBP2 | 221 |  | 22 | 221 |
|  | CSDE1 | 222 |  | 22 | 222 |
|  | DPH5 | 223 |  | 22 | 223 |
|  | CHMP2A | 224 |  | 22 | 224 |
|  | SLC9A3R1 | 225 |  | 22 | 225 |
|  | TOMM70 | 226 |  | 23 | 226 |
|  | DCTPP1 | 227 |  | 23 | 227 |
|  | ElectronT | 228 |  | 23 | 228 |
|  | FAR2 | 229 |  | 23 | 229 |
|  | WFS1 | 230 |  | 23 | 230 |
|  | PIGO | 231 |  | 23 | 231 |
|  | RETREG3 | 232 |  | 23 | 232 |
|  | UBXN8 | 233 |  | 23 | 233 |
|  | NLRX1 | 234 |  | 23 | 234 |
|  | TMEM97 | 235 |  | 23 | 235 |
|  | ERMP1 | 236 |  | 23 | 236 |
|  | TAPT1 | 237 |  | 23 | 237 |
|  | PIGS | 238 |  | 236 | 237 |
|  | GPAA1 | 239 |  | 23 | 238 |
|  | SLC30A6 | 240 |  | 23 | 239 |
|  | TMED5 | 241 |  | 23 | 240 |
|  | SCAP | 242 |  | 23 | 241 |
|  | BCS1L | 243 |  | 23 | 242 |
|  | NDFIP2 | 244 |  | 23 | 243 |
|  | DPY19L1 | 245 |  | 23 | 244 |
|  | F2RL1 | 246 |  | 23 | 245 |
|  | GHITM | 247 |  | 23 | 246 |
|  | ABCC1 | 248 |  | 23 | 247 |
|  | TMEM39B | 249 |  | 23 | 248 |
|  | ALG8 | 250 |  | 24 | 249 |
|  | Cul2 | 251 |  | 24 | 250 |
|  | ZYG11B | 252 |  | 24 | 251 |
|  | PPT1 | 253 |  | 24 | 252 |
|  | TIMM8B | 254 |  | 24 | 253 |
|  | THTPA | 255 |  | 24 | 254 |
|  | MAP7D1 | 256 |  | 25 | 255 |
|  | BRD2 | 257 |  | 25 | 256 |
|  | BRD4 | 258 |  | 25 | 257 |
|  | SLC44A2 | 259 |  | 25 | 258 |
|  | ZC3H18 | 260 |  | 25 | 259 |
|  | AP3B1 | 261 |  | 25 | 260 |
|  | CWC27 | 262 |  | 27 | 261 |
|  | RNAP | 263 |  | 27 | 262 |
|  | G3BP1 | 264 |  | 27 | 263 |
|  | G3BP2 | 265 |  | 27 | 264 |
|  | CSNK2A2 | 266 |  | 27 | 265 |
|  | CSNK2B | 267 |  | 27 | 266 |
|  | SNIP1 | 268 |  | 27 | 267 |
|  | FAM98A | 269 |  | 26 | 268 |
|  | PITRM1 | 270 |  | 26 | 269 |
|  | INTS4 | 271 |  | 26 | 270 |
|  | GGCX | 272 |  | 26 | 271 |
|  | FASTKD5 | 273 |  | 26 | 272 |
|  | FAM8A1 | 274 |  | 26 | 273 |
|  | ETFA | 275 |  | 26 | 274 |
|  | COQ8B | 276 |  | 26 | 275 |
|  | BZW2 | 277 |  | 26 | 276 |
|  | ATP6V1A | 278 |  | 26 | 277 |
|  | ATP1B1 | 279 |  | 26 | 278 |
|  | AASS | 280 |  | 26 | 279 |
|  | ACADM | 281 |  | 26 | 280 |
|  | AKAP8L | 282 |  | 26 | 281 |
|  | ANO6 | 283 |  | 26 | 282 |
|  | TUBGCP3 | 284 |  | 26 | 283 |
|  | TUBGCP2 | 285 |  | 282 | 283 |
|  | RTN4 | 286 |  | 26 | 284 |
|  | YIF1A | 287 |  | 26 | 285 |
|  | REEP5 | 288 |  | 26 | 286 |
|  | REEP6 | 289 |  | 26 | 287 |
|  | SLC30A9 | 290 |  | 26 | 288 |
|  | SLC30A7 | 291 |  | 26 | 289 |
|  | SLC25A21 | 292 |  | 26 | 290 |
|  | SAAL1 | 293 |  | 26 | 291 |
|  | AAR2 | 294 |  | 26 | 292 |
|  | TARS2 | 295 |  | 26 | 293 |
|  | STOM | 296 |  | 26 | 294 |
|  | PSMD8 | 297 |  | 26 | 295 |
|  | PMPCB | 298 |  | 26 | 296 |
|  | GOLGA7 | 302 |  | 28 | 299 |
|  | ZDHHC5 | 301 |  | 28 | 298 |
